# Imaging Appropriateness in Pediatric Radiology during COVID-19 Pandemic: A Retrospective Comparison with No COVID-19 Period

**DOI:** 10.3390/children8060463

**Published:** 2021-06-01

**Authors:** Giampiero Bottari, Giandomenico Stellacci, Davide Ferorelli, Alessandro Dell’Erba, Maurizio Aricò, Marcello Benevento, Giuseppe Palladino, Biagio Solarino

**Affiliations:** 1 Department of Interdisciplinary Medicine (DIM), Institute of Legal Medicine, University of Bari, Piazza G. Cesare 11, 70124 Bari, Italy; davideferorelli@gmail.com (D.F.); alessandro.dellerba@uniba.it (A.D.); marcello0835@gmail.com (M.B.); biagio.solarino@uniba.it (B.S.); 2 Department of Pediatric Radiology, Giovanni XXIII Pediatric Hospital, Via G. Amendola 207, 70126 Bari, Italy; gstellacci@me.com (G.S.); giuseppepalladino@hotmail.com (G.P.); 3 COVID-19 Management Crisis Unit, Giovanni XXIII Pediatric Hospital, Via G. Amendola 207, 70126 Bari, Italy; maurizio.arico@policlinico.ba.it

**Keywords:** COVID-19, pediatric emergency department, pediatric radiology department, diagnostic imaging, imaging appropriateness, healthcare policy

## Abstract

During the COVID-19 pandemic, the number of accesses to the Pediatric Emergency Department (pED) in Italy sharply decreased by 30%. The purpose of this study is to evaluate how this novel setting impacted on management of children with trauma, and the use and appropriateness of imaging studies in such patients at the pED. All imaging studies performed in patients with trauma at the pED of a tertiary children’s Hospital during the first wave of the COVID-19 pandemic (between March and May 2020) were reviewed, in comparison with a control time interval (March to May 2019). In the pre-COVID control era, 669 imaging studies documented bone fractures in 145/568 children (25.5%). In the COVID-era, 79/177 (44.6%) pediatric patients showed bone fractures on 193 imaging studies. Comparative analysis shows a 71% decrease in imaging studies, and the proportion of negative imaging studies (with no evidence of bone fractures) dropped in 2020 by 19% compared to the 2019 control era (*p* < 0.001). The sharp decrease of negative studies suggests that the rate of appropriateness was higher during COVID-era, suggesting some attitude toward defensive medicine in the previous control year, as a result of some degree of imaging inappropriateness. The impact of a pandemic on emergency medicine may offer a unique opportunity to revisit diagnostic and therapeutic protocols in pediatrics.

## 1. Introduction

By 9 March 2020, COVID–19 had been diagnosed in 8342 subjects in Italy. To reduce the spread of the pandemic, the Italian government progressively limited domestic mobility up to a complete ‘lockdown’ starting from 9 March, until 9 May 2020. People were allowed to leave their houses for very limited needs (mainly job or urgent medical needs) [1]. Therefore, urban circulation, as well as outdoor activities, were minimized, leading to an increase in children’s sedentary home life with health’s implications [2,3]. The locking down resulted, among other issues, also in a sharp decrease in admissions to the emergency departments (ED), including the pediatric ones (pED). The reduction, compared to the previous year, was in the range of 73–88% [4].

From this point of view, the COVID-19 pandemic represented an extraordinary ‘natural experiment’: such a sharp reduction of the admissions raised a question about the true appropriateness of the usual access to the pED in our non-pandemic life-style [5].

Technical progress of the medical imaging techniques resulted in a marked increase in the number of imaging studies performed. This has two drawbacks: not only do these studies have a cost, which builds an additional burden for the National Health System (NHS); also, inappropriate imaging studies are expected to harm children and adolescents because of the undue, increased carcinogenic risk due to radiation exposure [6,7].

To reduce the undue overuse of imaging in pediatric hospital care, at least two main lines can be pursued. On one side, better communication between the clinician and the radiologist; on the other side, better knowledge of the good and bad of imaging techniques. The use of guidelines may reduce the number of examinations by 20%, thus improving their appropriateness [8].

Better communication between specialists may definitely contribute to increase appropriateness of the diagnostic studies; to this issue, to reduce patient’s radio exposition, ‘daily communication’ between pediatric radiologists, ED pediatricians, radiology technicians, and nurses, is warranted. Regular pediatric radiology rounds may help to develop this cooperation. Hricak et al. demonstrated success in decreasing the number of CT examinations with ‘radiation awareness’ and ‘education’ through journal articles and lectures by pediatric radiologists [9]. German pediatricians were surveyed using a questionnaire, in which 14% of them stated that MRI causes radiation, and only 4% valued the dose-sparing potential of pediatric CT-protocols [10]. Furthermore, awareness of the guidelines is very helpful in reducing the overprescription of CT scans [11,12].

The COVID-19 pandemic should be regarded as an opportunity to introduce, or retain, good practices learnt on-the-go. Reassessing the appropriateness of medical practice may allow, in the field of pediatric imaging studies and radio-exposure, to balance between the increase justified by more technology available, and over-use deriving only from defensive medicine, where legal rather than medical reasons are in place [13,14].

This study aims to take profit of the pandemic experimental model to revisit the appropriateness of the current use of imaging studies in children with trauma seen in the pED of our third-level teaching children’s hospital in Apulia, Southern Italy.

## 2. Materials and Methods

The database of children and adolescents admitted to the pED of a public tertiary children’s hospital was analyzed, to identify the subset of patients admitted because of trauma. In these patients, the number, type, and results of imaging studies were recorded.

Data of patients admitted during the first wave of the COVID-19 pandemic (9 March to 9 May 2020) were compared with those of patients admitted during a historical pre-COVID control era (March to May 2019).

The study time interval fell within the national lockdown, held by the Italian Government between 9 March and 9 May 2020. The corresponding time interval (between 9 March and 9 May 2019) was taken as pre-COVID-19, control era.

The number and type of imaging studies performed, and the proportion of positive studies (i.e., those confirming the presence of traumatic bone lesions) were analyzed to evaluate the appropriateness of the requests.

The difference in the distribution of the variables was calculated by the chi-square test, Wilcoxon’s test, Fisher’s test, and the significance level was set at 0.05. The study received the approval of the local ethics committee.

## 3. Results

During the COVID-19 pandemic lock-down, fewer patients were admitted to the pED because of bone trauma than in the previous control era (177 vs. 568). Their demographics were not significantly different, except for a younger median age (6.3 vs. 7.9 years; *p* < 0.001).

In these 177 patients, 193 radiologic tests were performed, almost exclusively X-ray imaging studies (n = 181), with only 9 CT, and 3 ultrasound scans. Among them, 82/193 studies were positive for bone lesions (42.5%) in 79 patients (44.6%), while the remaining 98 patients (55.4%) had no evidence of bone lesions in the 111 imaging studies performed.

In the pre-COVID control era, 669 imaging studies had been performed on a comparable population: 631 X-ray, 19 CT, and 19 ultrasound scans. Of them, 156 (23.3%) were defined as positive, i.e., showed bone lesions, in 145 patients (25.5%). The remaining 513 were reported to be negative in the remaining 423 patients.

Imaging studies were focused on the appendicular skeletal system. During the COVID-period, imaging tests were significantly more frequently aimed at the upper limbs (n = 129), 22 at the lower limbs, 42 at the axial bones. During the previous year (2019), 329 studies were focused on the upper limbs, 229 on the lower limbs, and 111 on the axial system, including cephalic’ structures.

CT scan had a very limited use: 19 studies in 2019 (2 positives), and 9 during 2020 (only 1 positive).

Overall, during the pandemic, the number of pediatric patients admitted to pED because of trauma decreased by 68.8%; in the patients evaluated, the proportion of negative studies reduced by 19.1% (from 74.5% to 55.4%) (*p* < 0.001).

All result data are shown in Table 1.

## 4. Discussion

The COVID-19 pandemic may be considered, as already for AIDS, as a tremendous experimentum naturae. As suggested by several authors, the pandemic had a relevant impact on healthcare services, which were reorganized to cope with the ongoing outbreak [1,15]. In this retrospective observational study, we explored the hypothesis that not only the locking down of the population was associated with a reduction of accidents and trauma [16]; but also that the pandemic induced a reduction of parental attitude to take their children and adolescents to the pED for evaluation of suspected or minimal trauma [17]. The comparative analysis was performed on the events recorded during the months of lockdown (March to May 2020) versus the same calendar months of the previous year (2019).

Starting from the study population, during the pandemic the median age was 6.3 years, versus 7.9 years in the previous control era (*p* < 0.001). This might be explained by a lower proportion of patients with road accidents, more often adolescents, and a higher proportion of patients with home accidents, more likely to be young kids.

Reduction of visits at the pED by over three quarter during the lockdown was reported in a multicenter survey in Italy [18]. The sharp decrease (by 68.8%) of the primary accesses to our pED for trauma, observed during the lockdown regimen, results from the stop of social activities (schools, parks, gym) so that children and adolescents spent the entire day indoors, at home, reducing not only the risk of transmission of the common infectious agent but also the risk of traumatic injuries [14]. Moreover, the pandemic induced an emotional, although unjustified, fear to be exposed to SARS-COV2 infection when accessing the hospital. Pediatric practitioner daytime availability for phone consultations, may also have contributed to reduce ED overcrowding [19].

Moreover, the results also showed a significant decrease (by 19%) of the radiologic studies resulting in negative findings. This means that one in five imaging studies, which would have been undue, was omitted. This translates into fewer costs for the NHS, but also and even more important, in reduced exposure to irradiation for children and adults. What is the reason? We can hypothesize that in the pre-pandemic period the patients were older and with a higher proportion of lower limb injuries associated with football-playing trauma, in which bone fractures are less frequent. Another reason could be related to the lower number of visits at the pED, which allows physicians more time to dedicate to each patient, in a less stressed situation. This may have contributed to reduce the number of requests of imaging study. Thus, one indirect gift of the pandemic is that we may learn how to work at a better standard, with a higher level of appropriateness [20,21]. According to the RAND organization, the appropriateness defines the benefits related to assistance procedure (e.g., diagnostic or therapeutic) that should be “superior” to any negative consequence [22,23].

The appropriate use of radiologic examinations has been evaluated in the guidelines developed by the Royal College of Radiologists and in the ACR Appropriateness Criteria. They recommend a careful evaluation of radiologic examinations, evaluating the problem-solving of the clinical topic with “time”, deferring the radiology for 3 to 6 weeks [24]. The examination should not be performed unless the clinician provides cogent arguments for it [25,26].

The decision making phase for a better functioning diagnostic imaging technique could be enhanced by coordinated communication, where the pediatric radiologist could take on a primary role, because of the best interpretation of the clinical issues of the pediatric population, allowing a right education and coordination between the staff members [10]. Refined protocols might help in reducing the use of CT scan, rather than ultrasound. According to the ALARA (As Low As Reasonably Achievable) principle, full-time availability of the radiologist in a referral children hospital could help in this sense [8,27]. Teleradiology may contribute when allowing the radiologist to work in remote view, although its wide use would limit communication between health specialists [28].

This study has limitations. In order to assess the impact of COVID pandemic on pediatric population which were suffered traumatic injuries in daily life, we should have additional information about the actual number of injured children. Unfortunately, this information is not available.

## 5. Conclusions

This study demonstrated a decrement of both the rate of trauma pediatric patients and the radiological examinations, which were performed in our pED during spring 2020. The most important finding is that the proportion of negative studies reduced by 19.1%.

Overcrowding in the pED, and expectations of the family on imaging confirmation of the clinical diagnosis may result in defensive medicine. Imaging studies, when inappropriate, result in undue radiation exposure.

The impact of the pandemic on emergency medicine may offer a unique opportunity to revisit diagnostic and therapeutic protocols in pediatrics.

## Figures and Tables

**Table 1:** Descriptive results of the study population, during the COVID-19 era or during the pre-COVID control era.

	COVID-19 Era (2020)	Control Era (2019)	*p-*Value
**Study Population**	n = 177	n = 568	
Gender (male/female)	100/77	359/209	*p =* 0.109 *
**Age**			
Average	6.3	7.9	*p <* 0.001 *^*
Range	4 months–16 years	8 months–16 years	
**Imaging Studies**			
Total	193	669	
Positive (%)	82 (42.5%)	156 (23.3%)	*p <* 0.001 *
RX (%)	181 (93.8%)	631 (94.3%)	
Positive x-ray (%)	81 (44.7%)	151 (23.9%)	*p <* 0.001 *
US (%)	3 (1.5%)	19 (2.8%)	
Positive US (%)	0 (0%)	3 (15.8%)	*p =* 1 **
CT (%)	9 (4.7%)	19 (2.8%)	
Positive CT (%)	1 (11.1%)	2 (10.5%)	*p =* 1 **
**Radiological examinations for Anatomical District**			
Upper limbs (%)	129 (66.8%)	329 (49.2%)	
Positive (%)	65 (50.3%)	103 (31.3%)	*p =* 0.011 *
Lower limbs (%)	22 (11.4%)	229 (34.2%)	
Positive (%)	9 (40.9%)	40 (17.5%)	*p =* 0.043 *
Skull spine (%)	42 (21.8%)	111 (16.6%)	
Positive (%)	8 (19%)	13 (11.7%)	*p =* 0.312 *
**Patients with evidence of bone fracture**			
Positive (%)	79 (44.6%)	145 (25.5%)	*p <* 0.001 *

Significance level is set on 0.05; * Chi square test, ^ Wilcoxon’s test, ** Fisher’s test were adopted to evaluate *p*-values.

## Data Availability

The data presented in this study are available on request from the corresponding author.
